# Artificial intelligence for the detection of vertebral fractures on plain spinal radiography

**DOI:** 10.1038/s41598-020-76866-w

**Published:** 2020-11-18

**Authors:** Kazuma Murata, Kenji Endo, Takato Aihara, Hidekazu Suzuki, Yasunobu Sawaji, Yuji Matsuoka, Hirosuke Nishimura, Taichiro Takamatsu, Takamitsu Konishi, Asato Maekawa, Hideya Yamauchi, Kei Kanazawa, Hiroo Endo, Hanako Tsuji, Shigeru Inoue, Noritoshi Fukushima, Hiroyuki Kikuchi, Hiroki Sato, Kengo Yamamoto

**Affiliations:** 1 Department of Orthopedic Surgery, Tokyo Medical University, 6-7-1 Nishishinjuku, Shinjuku-ku, Tokyo, 160-0023 Japan; 2 Department of Preventive Medicine and Public Health, Tokyo Medical University, Tokyo, Japan

**Keywords:** Neurology, Pain, Diagnosis

## Abstract

Vertebral fractures (VFs) cause serious problems, such as substantial functional loss and a high mortality rate, and a delayed diagnosis may further worsen the prognosis. Plain thoracolumbar radiography (PTLR) is an essential method for the evaluation of VFs. Therefore, minimizing the diagnostic errors of VFs on PTLR is crucial. Image identification based on a deep convolutional neural network (DCNN) has been recognized to be potentially effective as a diagnostic strategy; however, the accuracy for detecting VFs has not been fully investigated. A DCNN was trained with PTLR images of 300 patients (150 patients with and 150 without VFs). The accuracy, sensitivity, and specificity of diagnosis of the model were calculated and compared with those of orthopedic residents, orthopedic surgeons, and spine surgeons. The DCNN achieved accuracy, sensitivity, and specificity rates of 86.0% [95% confidence interval (CI) 82.0–90.0%], 84.7% (95% CI 78.8–90.5%), and 87.3% (95% CI 81.9–92.7%), respectively. Both the accuracy and sensitivity of the model were suggested to be noninferior to those of orthopedic surgeons. The DCNN can assist clinicians in the early identification of VFs and in managing patients, to prevent further invasive interventions and a decreased quality of life.

## Introduction

Thoracolumbar vertebral fractures (VFs) occur very frequently in blunt traumas, and the accurate detection of VFs is crucial for the evaluation of patients with low back pain in primary care medicine. The mortality rate of patients with VFs is estimated to be 10–30% in the subsequent 1 year^1–3^. Substantial functional loss may occur after a VF^4,5^, and a delayed diagnosis may worsen the prognosis or may require invasive salvage surgery^6,7^. These individual issues associated with VFs may result in substantial economic burden to society^8^. Therefore, early diagnosis and treatment are essential for the prevention of not only functional loss but also the reduced quality of life of patients. Plain thoracolumbar radiography (PTLR) is an essential and widely used tool for the image evaluation of VFs. However, the sensitivity of PTLR for assessing VFs is not optimal. Errors and discrepancies with the results of radiology are estimated to be 10–30%^9–11^. Therefore, in clinical practice, clinical examination together with imaging analysis, such as computed tomography (CT) or magnetic resonance imaging (MRI) is used^12^. However, these evaluation methods are time-consuming or costly, and are not always available in the primary care setting, which is where most patients with low back pain are initially examined. Therefore, minimizing the diagnostic errors of VFs on PTLR, to prevent potential catastrophic results is of great importance. In light of these points, digital imaging systems may be useful not only for immediate and remote access^13^, but also for the possibility of automatic diagnostic procedures made by a deep convolutional neural network (DCNN)^14^. Image identification based on deep learning has been recognized to be a potentially effective diagnostic strategy, and has already become feasible^15^. The application of DCNN in medical image identification is expected to spread widely and rapidly; however, the accuracy of using DCNN for detecting VFs has not been fully investigated. In this study, we hence analyzed an automated VF diagnosis algorithm trained based on the DCNN, and investigated its performance compared with that of orthopedic surgeons, to test the hypothesis that DCNN is an adequate tool for the screening of VFs.

## Materials and methods

The collection and evaluation of patient image was made based on the latest Declaration of Helsinki, and also followed the ethical guidelines for clinical trials and the ethical guidelines for human-related medical research (Japanese Ministry of Health, Labor and Welfare). This study was approved by the Ethics Review Committee of Tokyo Medical University Hospital. Informed consent was obtained in the form of opt-out on the web-site of our department. Those who rejected were excluded.

### Study population

This was a retrospective study performed at a single medical center, which was a level 3 trauma center. After obtaining institutional review board approval, the data of demographic profiles, medical data, and medical imaging findings stored in a computerized database of patients who presented to our department with thoracolumbar VF from 2015 to 2018 were analyzed. Patients with low back pain or lumbar spinal canal stenosis without a past history of VF were also included as negative controls.

### Plain thoracolumbar radiography (PTLR) dataset

A total of 300 patients in the registry (150 patients with VF and 150 without VF) were included in the study. The demographic data of patients in the dataset are shown in Table 1. VF was diagnosed using the PTLR and MRI stored in a picture archiving and communication system (PACS). PTLR were obtained using BENEO-Fx (system number: MP95A9482001, Fujifilm co., Tokyo, Japan). The images used for diagnosis were obtained within 1 month after onset of symptoms. The size of the stored images of computed radiographs (CR) obtained from PACS as JPEG sized 960 × 720 pixels, and the color was 24-bit grayscale. Each patients was assigned a serial number and deidentified in both the images and registry. Patients with a VF of grade 1 using the semiquantitative grading method^16^ were excluded, because grade 1 VF is considered to be difficult to distinguish from vertebral deformities^17,18^. Patients with 2 or more VFs were also excluded. After the PTLR datasets were established, images were initially labeled as a VF or negative by spine surgeons, where the patients were diagnosed as having a VF if both vertebral collapse on PTLR and typical signal changes on MRI of low T1, high T2, and high short-tau inversion recovery were observed. Each image was reviewed by a registered spine surgeon for the preciseness of the labeling and quality of the images and put into DCNN described below without any processing.

**Table 1:** Demographic data of the patients.

	VF	Without VF	*p* value
Number of patients	150	150	
Age (years)	69.1 ± 1.4	65.4 ± 1.4	0.73
Sex, female (%)	92 (61.3%)	91 (60.7%)	0.96

*VF* vertebral fracture.

### Development of the algorithm

**Figure 1:**
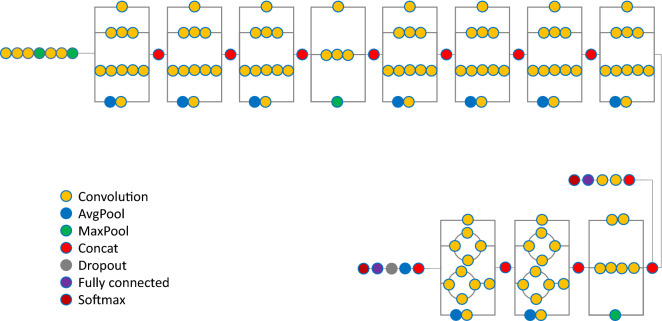
Representative of Visual Recognition V3 model.

**Figure 2:**
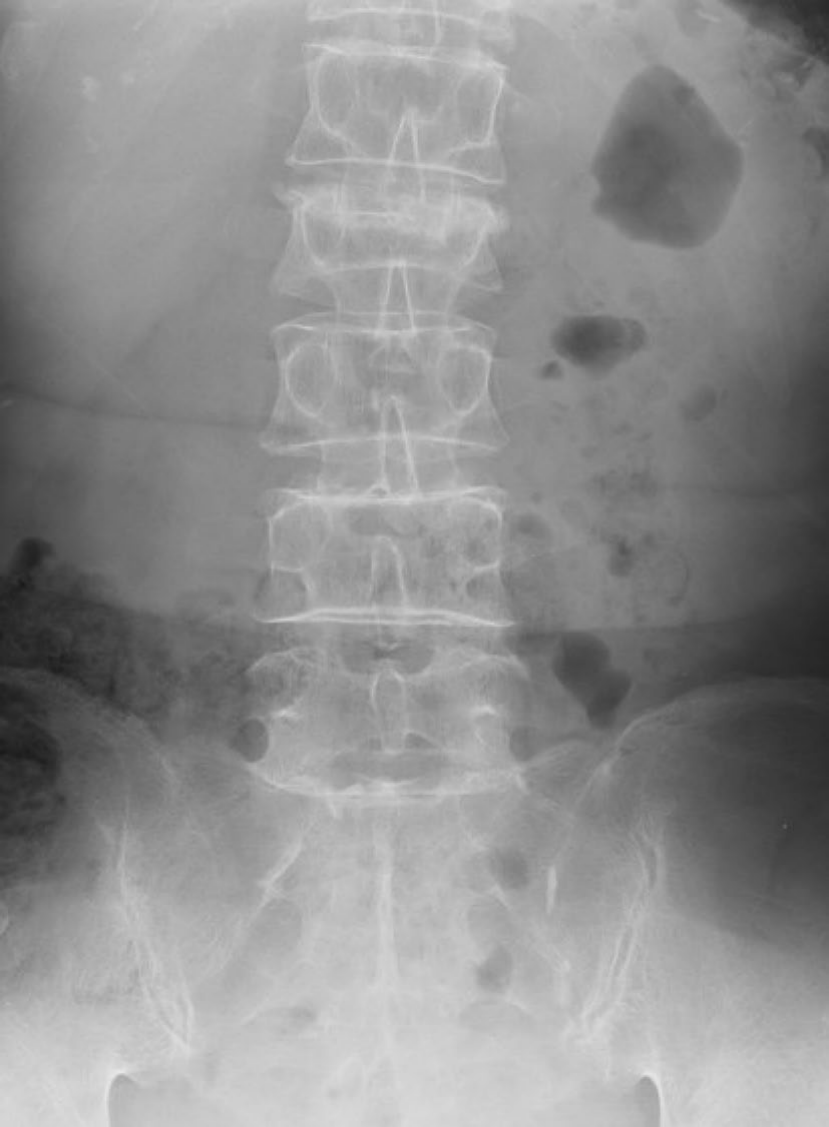
Representative of antero-posterior view of PTLR of a patient with VF. The image shows VF on L3 (arrow).

**Figure 3:**
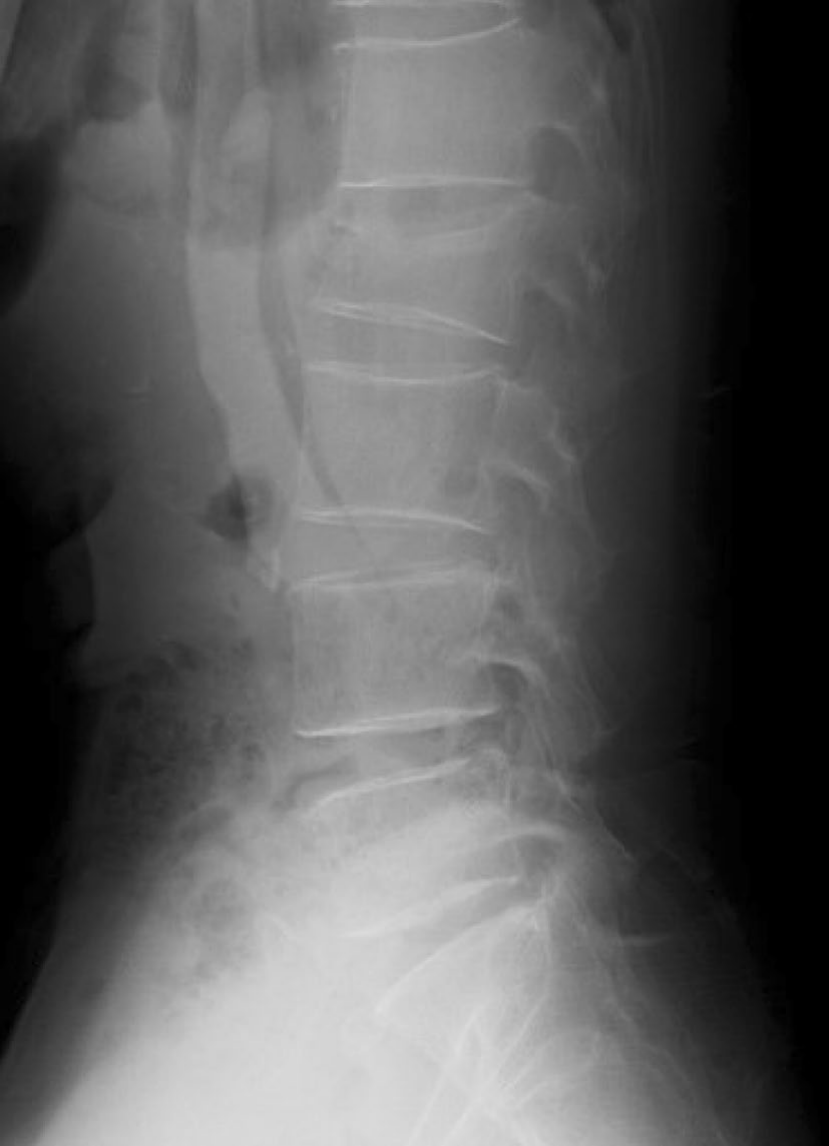
Representative of lateral view of PTLR of a patient with VF. The image shows VF on L3 (arrow).

**Figure 4:**
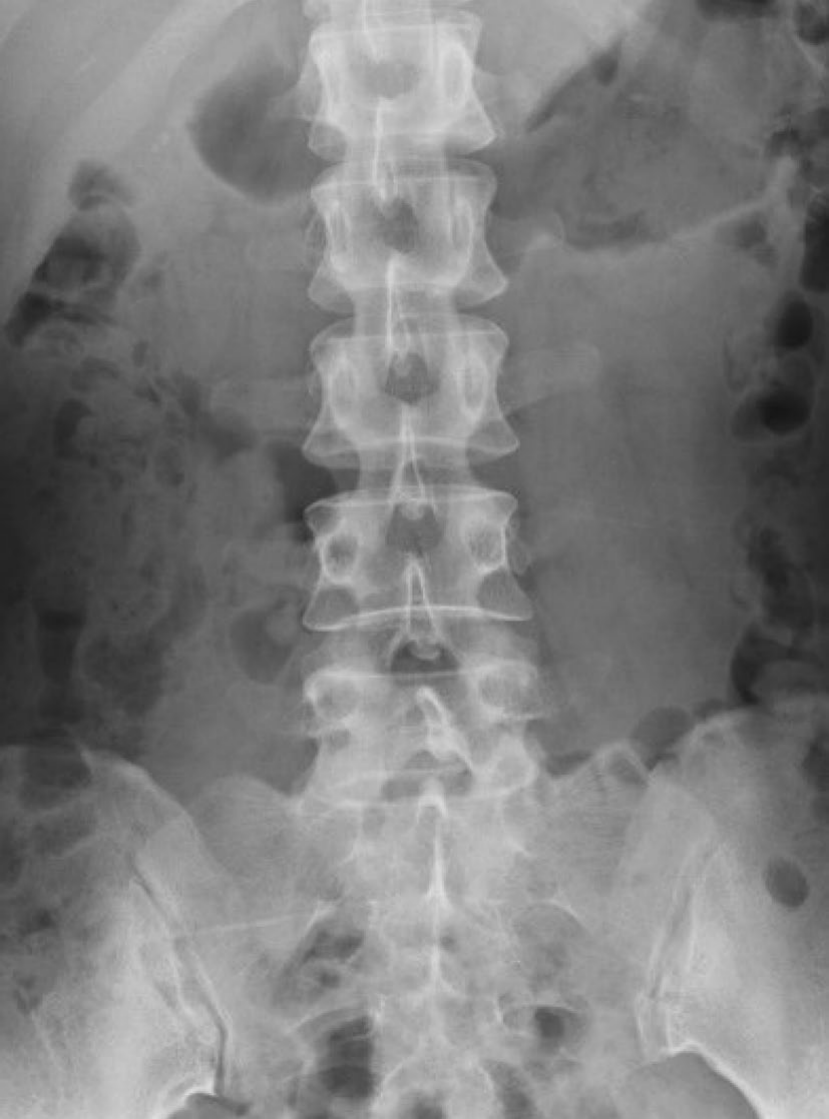
Representative of antero-posterior view of PTLR of a patient without VF.

**Figure 5:**
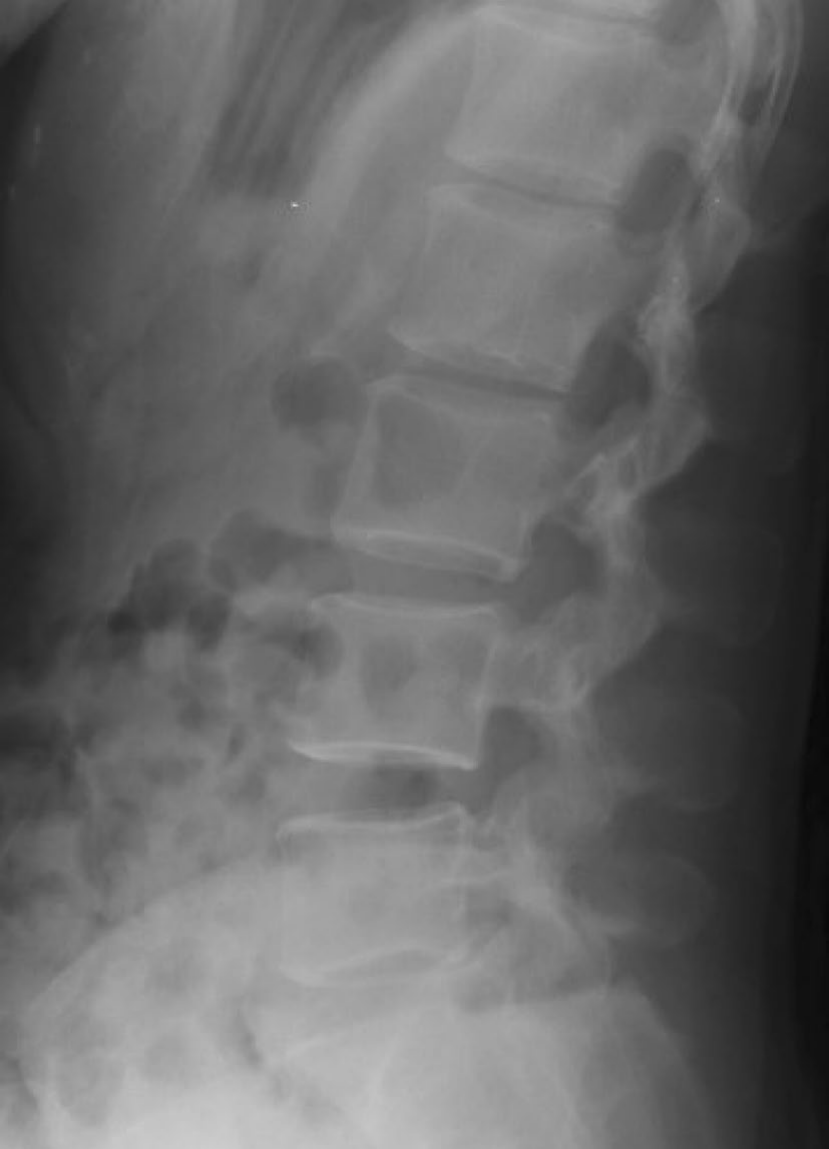
Representative of lateral view of PTLR of a patient without VF.

DCNNs are widely used in medical image recognition^19^. DCNNs are machine-learning algorithms used by artificial intelligence. The basic concept is to use pixel values from a digital image as inputs, using techniques such as convolution and pooling on each layer, and to adjust the weights in the neural network according to the difference between the output and true label. After a significant amount of imaging input is used as training material, the weights in the neural network are adjusted to fit the problem. The tools of Classify Images of Watson Studio based on free account of Visual Recognition V3 imported into Watson Studio published on IBM cloud was used as the structure of the neural network in this study (https://www.ibm.com/jp-ja/cloud) (Fig. 1). The input images were tested as the set of the antero-posterior and lateral PTLR on the JPEG file for each patient, and resized to 512 × 512 pixels with an 8-bit grayscale color to reduce the complexity and computation (Figs. 2, 3, 4, 5). K-fold cross validation (k = 5) was used for the evaluation of VFs. The hyperparameters such as batch size and optimized epochs were assigned automatically by Visual Recognition V3.

### Algorithm evaluation

The trained model was tested to analyze its accuracy in identifying VFs. The probability generated by the VF model was analyzed using a receiver operating characteristic (ROC) curve and the area under the curve (AUC). A confusion matrix was also calculated using a cutoff level of probability of 0.80 for VFs^20^, in which 0.80 was also predicted using the ROC table. In addition, orthopedic doctors were divided into 3 groups, i.e., orthopedic residents (n = 20), board certified orthopedic surgeons (n = 24), and board certified spine surgeons (n = 9). The diagnosis made by each orthopedic doctor was retrieved from the medical records. Therefore, the diagnosis of the physician was made not only by image diagnosis of PTLR but also by physical findings. Gold Standards of diagnosis of VF was determined by spine surgeons with the findings of typical signal changes on MRI of low T1, high T2, and high short-tau inversion recovery.

### Statistical analysis and software

All statistical analyses were performed using JMP 14.0 (SAS Institute, Inc., Cary, NC, USA). Continuous variables were analyzed using the Student *t*-test after determining that the data followed a parametric distribution using the Shapiro–Wilk normality test (in which *p* > 0.05 suggested that the data was from a normal distribution), and categorical variables were analyzed using the Fisher exact test. We compared the trained models and orthopedic doctors using the accuracy, sensitivity, specificity, and 95% confidence intervals (CIs) of each parameter. The McNemar test was used to evaluate the noninferiority of the accuracy, sensitivity, and specificity of the DCNN compared with orthopedic residents, orthopedic surgeons, and spine surgeons^21^. The kappa coefficient was calculated between the diagnosis made by the DCNN and the physicians.

## Results

**Figure 6:**
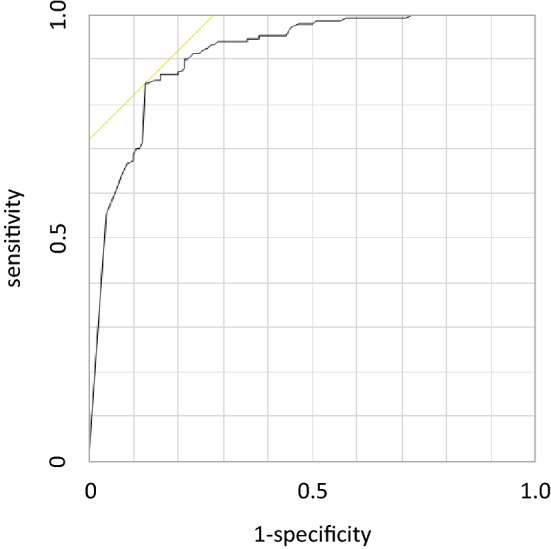
ROC curve of the model. The ROC curve of the prediction probability is shown as a black line. A yellow line shows the tangent line of the curve. The model achieved an AUC of 0.91 (95% CI 0.96–1.00).

The accuracy, sensitivity, and specificity of the models are shown in Table 2. The ROC curve of prediction probability is shown in Fig. 6. The model achieved an AUC of 0.91 (95% CI 0.96–1.00).

**Table 2:** Predictive values for the diagnosis of VF.

	Value (%)	95% CI	*p* value
**DCNN**			
Accuracy	86.0	82.0–90.0	1.00
Sensitivity	84.7	78.8–90.5	1.00
Specificity	87.3	81.9–92.7	1.00
**Orthopedic residents**			
Accuracy	77.5	64.7–90.3	0.08
Sensitivity	72.4	56.7–88.1	0.02
Specificity	90.9	70.7–100	0.56
**Orthopedic surgeons**			
Accuracy	88.0	82.3–93.6	0.72
Sensitivity	77.5	67.8–87.1	0.31
Specificity	100	100	–
**Spine surgeons**			
Accuracy	98.4	95.5–100	< 0.01
Sensitivity	96.0	89.1–100	0.01
Specificity	100	100	–

*DCNN* deep convolutional neural network, *VF* vertebral fracture, *95% CI* 95% confidence interval.

The overall accuracy, sensitivity, and specificity of orthopedic residents, orthopedic surgeons, and spine surgeons are shown in Table 2. The McNemar test demonstrated that the DCNN had higher sensitivity compared with orthopedic residents (*p* = 0.02), but that accuracy, sensitivity of DCNN was not statistically significant compared with orthopedic surgeons (*p* = 0.72, *p* = 0.31, respectively). The performance of spine surgeons was significantly higher in accuracy, sensitivity, and specificity than the DCNN. The kappa coefficient of DCNN was calculated as 0.36 (*p* = 0.01) for orthopedic residents, 0.48 (*p* < 0.01) for orthopedic surgeons, and 0.66 (*p* < 0.01) for spine surgeons.

Twenty-seven out of the 300 patients (26 patients with VF, 1 patient without VF) were misdiagnosed by the physicians. In these cases, DCNN successfully diagnosed VF or not except in 1 patient with VF. There was only 1 case with VF that was misdiagnosed by both the DCNN and the physicians.

## Discussion

Our results demonstrated that a DCNN can be trained to identify VFs within image datasets with high sensitivity (84.7%) and specificity (87.3%). At present, although VFs are common, their diagnosis and treatment may be difficult, particularly in some clinical situations, such as in primary care, emergency medicine, or remote rural areas^22–24^. With the assistance of the DCNN, VFs can be detected immediately with a high accuracy rate (86.0%), which may be as high as the detection accuracy of expert’s clinical diagnosis. This DCNN may help primary physicians to avoid misdiagnoses, as well as subsequent unfavorable events: Missed fractures required surgery in 50% patients^25^. Early detection and treatment are crucial for patient survival and the preservation of spinal function. The delayed diagnosis of VF may result in a poor prognosis and even an increased risk of death after a few years^26–28^. Therefore, the accurate and immediate detection of VF is crucial for preventing mortalities and for favorable medical outcomes.

DCNN for image diagnosis in orthopedics is becoming widely used with its advancements. The ability to immediately classify a radiograph will most likely have a major effect on clinical orthopedic image diagnoses. Some previous studies have reported the possibility of DCNN in detecting bone fractures^29,30^. These findings shed light on the possibility of the clinical use of DCNN in orthopedics. However, the actual outcome of detecting a fracture still remains unknown, because most of the available fracture classifications lack prognostic value^31^.

In this study, DCNN detected VFs with a satisfactory accuracy. One of the strengths of our model may be the nature of using PTLR. There have been various techniques of the diagnosis of VF, but a gold standard has yet to be established^32–34^. The correct diagnosis rate for incident vertebral fractures by PTLR was estimated significantly low^35,36^, and inferior compared to that of CT^37^. However, PTLR is economic examination, which cost one fourth of MRI in our country. Furthermore, estimated figures for radiation dose for CT are 19.4 mSV for thoracolumbar spine CT, on the other hand, that of PTLR are 1.0–6.6 mSV^38,39^. These issue may shed light on the efficiency of DCNN. However, DCNN was unable to identify VFs in 21 patients in this study, and several misdiagnoses are considered owing to this simple preparation without clinical information. A large number of cases will simply improve the sensitivity of DCNN; however, there is expected to be both cases that are suitable for learning and those that are not, and future studies should be performed to solve this issue. In addition, we excluded old VFs in this study. In the clinical situation, some radiological VFs can be diagnosed as old VFs, from findings such as fused vertebrae, a bridging callus, or a vertebral cleft; however, we also encountered cases of radiological VFs, in which it was difficult to determine whether they were fresh or old. This exclusion is considered to be a limitation of our model, and hence the specificity might be increased in the clinical diagnosis made by physicians. In the progressive algorithms, these old VF should be included in anticipation of the clinical use. However, the deep learning algorithm is considered to be sufficient to achieve an accuracy level that is compatible with the accuracy of clinical diagnosis made by physicians to date.

This study has several limitations. One fundamental limitation is that we did not evaluate the functional prognosis of the VFs. Owing to the nature of DCNNs providing binary classification, the neural network provided only an image diagnosis with or without VF where level of fracture or instability of fracture were not evaluated. This issue might be similar to the limitation of diagnostic value of PTLR where CT or MRI might provide more valuable information to determine the need for surgical intervention^40^. Therefore, the diagnosis made by DCNN should be re-evaluated by surgeons, to confirm the diagnosis or to determine the treatment suitable for the patient. On the other hand, the diagnosis made by physicians were retrieved from the medical records, which included important information regarding the decision-making process, such as pathological onset, degree of pain, present history, medical background, and physical examination, and such information contributed to the high specificity rate of the physicians. The lack of such information in the DCNN may be unfavorable for decision-making. This disadvantage appears to emphasize the importance of the diagnosis by DCNN. The second limitation is the process of diagnosis. The process used by DCNN to learn the features of VF remains unknown. It hence remains unclear as to what properties and dataset volume are suitable for analysis. Features that were most useful for DCNN might be those previously unknown to or ignored by humans. This fact is confusing in regard to the authenticity of the diagnosis. However, DCNN may also be more efficient in its combined recognition of bone morphology, comminution, and bone quality, apart from traditional orthopedic measures. In addition, the detection of other lesions on the image such as coincidental tumor, which is essential for routine diagnosis, was not included in this study. And the insufficient fractures related to osteoporosis and pathological fractures secondary to metastasis were not present in the training set. These detection of traumatic VFs is not sufficient for clinical use, because there are red flags of low back pain other than VFs. Despite these limitations, we consider our diagnosis method to be useful for future practical applications. Our present results may be useful in the primary care setting as a method for the fast screening of VFs, even in situations in which experts are not available.

## Conclusion

To identify VFs on PTLR, an algorithm trained by a DCNN achieved excellent performance, with high accuracy and sensitivity. DCNN may have a potential for expansion as a screening tool to assist clinical physicians in identifying VFs. Further investigations with heterogenous cohort such as healthy controls or osteoporotic subjects are needed for the advancement of the model.
